# Mid-term Follow-up of the Transcatheter Closure of Perimembranous Ventricular Septal Defects in Children Using the Amplatzer

**Published:** 2015-10-27

**Authors:** Mehdi Ghaderian, Mahmood Merajie, Hodjjat Mortezaeian, Mohammad Yoosef Aarabi Moghadam, Akbar Shah Mohammadi

**Affiliations:** 1 *Child Growth and Development Research Center, Isfahan University of Medical Sciences, Isfahan, Iran. *; 2 *Shaheed Rajaie Cardiovascular, Medical and Research Center, Iran University of Medical Sciences, Tehran, Iran.*

**Keywords:** *Heart septal defect*, *ventricular*, *Heart defects*, *congenital*, *Septal occluder device*

## Abstract

**Background:** The ventricular septal defect (VSD) is the most common form of congenital heart defects. The purpose of this study was to evaluate the results of the early complications and mid-term follow-up of the transcatheter closure of the VSD using the Amplatzer VSD Occluder.

**Methods: **Between April 2012 and October 2013, 110 patients underwent the percutaneous closure of the perimembranous VSD. During the procedure, the size and type of the VSD were obtained via ventriculography. A device at least 2 mm larger than the VSD diameter measured via ventriculography was deployed. The size of the VSD, size of the Amplatzer, and device-size to VSD-size ratio were calculated. After the confirmation of the suitable position of the device via echocardiography and left ventriculography, the device was released. Follow-up evaluations were done at discharge as well as at 1, 6, and 12 months and yearly thereafter for the VSD occlusion and complete heart block.

**Results:** The study population comprised 62 females and 48 males. The mean age and weight of the patients at procedure were 4.3 ± 5.6 years (range: 2 to 14) and 14.9 ± 10.8 kg (range: 10 to 43). The average device size was 7.0 ± 2.5 mm (range: 4 to 14). The VSD occlusion rate was 72.8% at the completion of the procedure and rose up to 99.0% during the follow-up. The most serious significant complication was complete atrioventricular block, which was seen in 2 patients. The mean follow-up duration was 10.9 ± 3.6 months.

**Conclusion: **The transcatheter closure of the perimembranous VSD was a safe and effective treatment with excellent closure rates in our study population. This procedure had neither mortality nor serious complications.

## Introduction

The ventricular septal defect (VSD) is the most common form of congenital heart defects. It constitutes approximately 30% of all congenital heart diseases.^1^^, ^^2^ With the advent of echocardiography and increasing experience of the operators, the recognition of the VSD has increased to 5 / 1000 live births.^3^^, ^^4^ Multifactorial and genetic causes are postulated to be responsible for the creation of the VSD. It is the most common congenital heart disease associated with trisomy 21, 13, and 18. For the description of the VSD, multiple classifications have been proposed. In the simplest classification, the VSD is divided into perimembranous, muscular, and inlet types. Small VSDs have neither significant pulmonary overload nor overcirculation. Medical treatment, surgical therapy, and percutaneous closure of these small defects are not suggested, and the outcome of these patients is excellent. For moderate or larger defects that result in an increased pulmonary flow, the surgical or interventional approach is recommended. 

The Amplatzer VSD Occluder was added to the interventional cardiology armamentarium in 1999.^5^^, ^^6^ The Amplatzer Membranous VSD Occluder is an asymmetrical double-disc device that was first described for the closure of membranous defects in 2000 and had its first clinical experience published in 2002.^7^^, ^^8^ The salient advantages of the use of this device are shorter hospital stay and avoidance of sternotomy and cardiopulmonary bypass. One of the most serious complications of this method, however, is the creation of atrioventricular conduction block (AVB). The follow-up studies of the patients who have had VSD closure with the Amplatzer VSD Occluder are limited to fewer than 10 years, in contrast to the surgical approach, which is now approaching to more than 60 years. Early and delayed AVBs have been described in fewer than 1% of patients after the surgical closure of the isolated VSD, whereas this complication ranges from 3% to 20% after the interventional approach and percutaneous closure.^7^^-^^9^ The immediate and short-term results of the transcatheter closure of the VSD with the Amplatzer VSD Occluder have been well documented.^10^^, ^^11^

We sought to evaluate the early and mid-term follow-up results of the transcatheter closure of the VSD using the Amplatzer VSD Occluder in children. 

## Methods

The study protocol was approved by the Ethics Committee of Iran University of Medical Sciences. In this prospective study, all patients scheduled for the transcatheter closure of the VSD between April 2012 and October 2013 in Rajaie Cardiovascular, Medical, and Research Center were included. There were 110 patients, comprised of 62 females and 48 males. Written informed consent was obtained from the patients’ parents after they were provided with a comprehensive explanation about the procedural details and the advantages and possible complications. The patients were examined in accordance with a standard echocardiographic protocol. All the patients underwent transthoracic echocardiography (TTE), performed with a GE Vivid 3 machine. TTE included M-mode, two-dimensional, and color Doppler examinations. The standard technique was used to obtain the measurements in a quiet, wakeful, and non-sedated state for the older children and a sedated state for the younger children. The ejection fraction was estimated using M-mode and Simpson in the parasternal long- and short-axis views as well as in the apical four-chamber view, with two-dimensional and color Doppler being employed in all the views. The size and type of the VSD were examined in the standard four-chamber, five-chamber, and parasternal long- and short-axis views. 

The criterion for inclusion in our study was the echocardiographic or clinical evidence of a significant left-to-right shunt through the VSD (> 1.5 / 1). A significant left-to-right shunt was considered to be a VSD, and one or more of the following data were found: 1) prominent cardiomegaly on standard chest X-ray; 2) left atrial enlargement, defined as a left atrial to aortic ratio > 1.5 in the long-axis parasternal view examination; 3) left ventricular overload and enlargement in echocardiography, defined as a left ventricular end-diastolic diameter ≥ 2 standard deviation (SD) for the body surface area; and 4) symptoms, including frequent respiratory infections (six events during a year) and/or failure to thrive. Infants were selected for transcatheter VSD closure if they had a minimal weight of 10 kg. The exclusion criteria were as follows: 1) VSD associated with any other congenital heart disease which could be corrected surgically; 2) significant cardiac and non-cardiac comorbidities and anomalies that could impact the clinical outcome of the VSD closure as well as evidence of severe and prolonged congestive heart failure (After modifying the underlying diseases of these patients and medical treatment, we enrolled them in the study.); 3) VSDs with severe pulmonary artery hypertension and a right-to-left shunt or pulmonary vascular resistance > 8 Woods units; 4) weight < 10 kg; 5) perimembranous VSDs closer than 5 mm to the aortic valve and prolapse of an aortic cusp; 6) sepsis; and 7) contraindication to antiplatelet therapy. 

The percutaneous closure of the VSD was performed under general anesthesia. Only the first-generation VSD Occluder was used in our study. The femoral vein and the arterial line were obtained. The patients were given 100 IU/kg of heparin in two divided doses (50 IU/kg first and 50 IU/kg half hour later) to maintain an activated clotting time > 200 seconds after the catheterization of the femoral artery. Prophylactic intravenous antibiotic with 30 mg/kg of cefazolin was administrated at the beginning of the procedure and by two subsequent doses every 8 hours during the following 24 hours. The procedure was performed under fluoroscopic and TTE control. Previously we had used transesophageal echocardiography (TEE) for our procedures; however, our further experience led us to utilize TTE for the VSD closure. Standard right and left cardiac catheterization and angiography were performed. During the cardiac procedure, angiographic and catheterization data, including the size and type of the VSD and its position to the aorta, were obtained via ventriculography in the left anterior oblique (LAO) cranial view in different degrees and the right anterior oblique (RAO) view. The prolapse of the aortic cusps was identified via LAO aortography in the ascending aorta. The right ventricular pressure, pulmonary artery pressure, and fluoroscopy time were identified via right and left cardiac catheterization. 

A device at least 2 mm larger than the VSD diameter measured by ventriculography was chosen. Totally, 28 Amplatzer Ductal Occluders and 82 Muscular VSD Occluders were used for the closure of the perimembranous VSDs. The other VSDs, i.e. apical or muscular VSDs, are not discussed here. Subsequently, a 0.035-inch Terumo Glide Wire was placed across the VSD using a 4- or 5-French curved end-hole catheter (Judkins Right Coronary Catheter, Cobra) from the left ventricle into either branch pulmonary artery or superior or inferior vena cava. The next step was to snare the wire and exteriorize it to the femoral vein and establish an arteriovenous loop. Over this wire, an appropriately sized delivery sheath was advanced from the femoral vein by the standard protocol all the way until the tip of the sheath was in the descending aorta. Thereafter, in order to measure the VSD diameter, a ventriculogram in the best possible view was obtained. The device was loaded under a blood/saline mixture. The device was, subsequently, attached to the delivery sheath and advanced to the tip of the sheath during fluoroscopy. The device was deployed in accordance with the standard protocol in the VSD, and ventriculography was done. After the confirmation of the suitable position of the device by echocardiography and left ventriculography, the device was released. 

Complete blood cell count and chest X-ray were performed 4-6 hours later to detect early complications such as occult bleeding and pulmonary complications. Additionally, an echocardiographic examination was performed to rule out pericardial effusion. Urine analysis to exclude hemolysis was done the day after the procedure. Vital signs monitoring was conducted during the first 24 hours. Moreover, 24-hour electrocardiographic (ECG) Holter was done the day after the procedure for dysrhythmia and heart block evaluation. All the patients received 1 mg/kg of clopidogrel for 3 days and 0.5 mg/kg for one month, 3 mg/kg of Acetylsalicylic Acid (ASA) for 6 months, and 50 IU/kg of heparin every 6 hours for the first day. All the patients were discharged after ECG Holtering. Endocarditis prophylaxis was done for 6 months if necessary and was discontinued at 6 months’ follow-up if the defect was completely occluded. ECG was done in routine visits at follow-up. All the patients had complete TTE studies before discharge as well as at 1, 6, and 12 months and then yearly thereafter.

The data are expressed as frequencies or percentages for the nominal variables. The continuous variables are expressed as mean ± SD. For the statistical analyses, the statistical software SPSS version 18.0 for Windows (SPSS Inc., Chicago, IL) was used.

## Results

Table 1 depicts the demographic, general, clinical, and analytical data of the patients. The clinical characteristics of the patients, e.g. age, sex, and weight, were recorded.

During the study, with the intention to close the VSD percutaneously, all the patients who had the inclusion criteria were sent to our catheterization laboratory. The defects were successfully closed in 109/110 (99.09%) patients. Device embolization occurred in none of the patients. In one patient during the procedure, approximately 10 cm of the distal segment of the wire was broken in the aortic arch and this segment was embolized into the carotid artery. After this section was effectively re-snared via the arterial line, the VSD closure was performed successfully. In 2 patients, the devices were too small to close the defect and during the procedure the devices were pushed into the right ventricle; therefore, larger devices were implanted. We had no mortality in our study. One patient had complete heart block during the procedure, which could not be reversed by conservative treatment. In this patient, the defect was not closed; however, the device was excluded and the patient was referred for surgical correction. This patient had the lowest weight among our patients. One patient had complete heart block 2 weeks later: for this patient, pacemaker implantation was performed. Complete heart block did not occur any more in our study patients during the follow-up period. We had no major arterial complications such as massive bleeding or femoral arteriovenous fistula in our study. Mild thrombosis of the right or left femoral arteries and weak pulse occurred in 10 patients after the procedure. Heparin infusion was used successfully in 8 patients during the first 24 postprocedural hours; the patients' pulses returned to normal during the first hours. Streptokinase intravenous infusion conferred a successful and complete turn of pulse without sequel in the other 2 patients. None of the patients needed further treatment or femoral artery thrombectomy. In all the patients, 5-French sheaths for the arterial lines and different sizes for the venous lines were used. All of these patients were less than five years old and weighed less than 15 kg. Seven patients had a small second VSD in addition to the first defect; following device implantation, 6 of these patients had no residue and one patient had mild residue at follow-up. In 9 patients, transient arrhythmias such as tachycardia, bradycardia, and bundle branch block developed during different maneuvers of the catheter or the wire. The procedure was stopped in these patients, and sinus rhythm quickly returned in all of them. None of our patients developed infectious endocarditis, and no mortality was reported. The left ventricular dimensions returned to normal in all the patients. In the patients with failure to thrive, their growth returned to the normal pattern. Patients with recurrent pneumonia had no significant recurrences. Follow-up at these time points (i.e. one month to 24 months) showed no significant complications such as new aortic regurgitation, device embolization or malposition, thrombus or clot formation, hemolysis, and thromboembolism. 

Angiography at the end of the procedure demonstrated complete occlusion in 72.8% of the patients immediately after the completion of the procedure; it rose up to 89.2% at discharge and 99.0% during the follow-up period. 

**Table 1:** Patients characteristics*

Variant	All Patients (n=110)	Male (n=48)	Female (n=62)	Range (All Patients)
Age (y)	4.3±5.6	5.2±6.7	3.4±4.8	2-14
Weight (kg)	14.9±10.8	16.3±14.2	14.2±13.2	10-43
VSD size (mm)	4.7±1.8	4.6±1.6	4.9±1.8	3.1-7.2
PA pressure (mm Hg)				
Systolic	31.3±14.2	34.2±16.1	30.2±12.1	20-57
Diastolic	16.2±8.4	17.8±9.2	15.3±7.1	10-40
Mean PA pressure (mm Hg)	22.6±6.4	25.2±7.1	21.6±6.5	14-45
Qp/QS	2.3±0.7	2.1±0.9	2.4±0.8	1.7- 4.8
Fluoroscopy time (min)	14.4±9.7	15.1±10.1	14.9±9.4	7.8-35.0
Total angiography time (min)	42±12.3	42.5±12.7	41.1±11.9	31-70
Size of Amplatzer (mm)	7.0±2.5	7.1±2.3	6.8±2.8	4–14
VSD size at ventriculogram	5.2±2.3	5.6±2.5	5.0±2.1	3-12
Device size/VSD size ratio	1.2±0.3	1.3±0.4	1.1±0.3	1.1-1.7

VSD, Ventricular septal defect; QP/QS, Pulmonary blood flow to systemic blood flow; PA, Pulmonary artery

* Data are presented as mean±SD or minimum-maximum

## Discussion

This study reports the mid-term (10.9 ± 3.6 months, range: 1 to 24 months) results of follow-up in 110 children who underwent the transcatheter closure of the VSD with the Amplatzer VSD Occluder. The VSD is the most common congenital heart disease.^1^^, ^^2^^, ^^12^ Patients with moderate to large VSDs and volume overload of the left ventricle due to these defects require the closure of the defect to prevent cardiac complications.^13^^, ^^14^ The interventional approach in comparison with the surgical approach for the treatment of congenital heart diseases is better appreciated by pediatric patients and their parents because of shorter lengths of hospitalization, avoidance of sternotomy, and fewer complications. The Amplatzer VSD Occluder has been proved to have all of the mentioned advantages. 

The transcatheter closure of the VSD has rare complications such as device embolization to the right or left side of the heart, AVB, and femoral artery thrombosis. Late complications are rarer, and AVB, late device embolization, and hemolysis are described in the literature.

In selected patients who had at least a 5-mm distance between the superior rim of the defect and the aortic cusps, the Amplatzer VSD Occluder was successfully deployed via the retrograde approach.^8^ The use of specific Amplatzer VSD Occluders for the perimembranous VSD has expanded the indications for percutaneous closure to cases with only 1 to 2 mm between the aortic valve and the defect, and the rate of successful closure is between 90% and 100%.^15^^-^^17^ The results of our study of the transcatheter closure of the perimembranous VSD with the Amplatzer VSD Occluder were excellent: we achieved an occlusion rate of 99.1% at follow-up, which is comparable with other reported results.^15^^-^^17^ Although some patients had a residual shunt at the end of the procedure in our study, only one (0.9%) patient had a small residual shunt during the follow-up period. None of our patients developed aortic valve regurgitation or valve injury. 

One of the common morphologic variations in the perimembranous VSD is the presence of aneurysm formation in the ventricular septum. This aneurysm formation was found approximately in half of our patients. During the procedure, we tried to close the true anatomic defect with the best appropriate device, depending on our judgment of the patients and their VSD sizes. In patients with small aneurysms, the device was able to cover the defect and the aneurysm together. In cases of large defects and large aneurysms, to avoid using an oversized device and to close the true defect, we implanted the devices within the aneurysms themselves. At follow-up during this time, TTE demonstrated a small residual shunt in one (0.9%) patient.

The occurrence of complete AVB is the only serious and worrisome complication of interventional perimembranous VSD closure. In patients treated for percutaneous VSD closure, the occurrence of complete AVB can be a late complication compared with the surgical approach, in which complete AVB usually appears early after the operation.^8^ The rates of complete AVB reported in the literature vary between 0 and 5%,^15^^-^^18^ although a higher rate was reported in one study elsewhere.^9^ There are no data on the mechanisms involved in the occurrence of complete AVB following the interventional approach and the percutaneous closure of the perimembranous VSD. Complete heart block has been reported to occur at any time from a few minutes to months after successful and uncomplicated procedures.^18^^, ^^19^ Currently, because of the high reported incidence of complete heart block by this approach, the device is not approved for the perimembranous closure of the VSD; and device modification, which may result in a reduction in external forces exposed onto the conduction system, is needed. The occurrence of complete AVB is related mostly to the conduction system around the margins of the defect; accordingly, both device implantation and surgical approach may interfere with atrioventricular conduction and induce complete heart block. Larger devices may cause more external forces and direct compression trauma or promote an inflammatory reaction and scar formation in the conduction tissue and give rise to the occurrence of complete AVB. This complication occurred in one (0.9%) patient with a perimembranous VSD in our series during the procedure, which disappeared immediately when we removed the device, and in another (0.9%) patient after the procedure, which necessitated pacemaker implantation. These rates rank among the lowest reported in the literature. 

In our series, the median follow-up was 10.9 months, and complete AVB was seen in one patient. Some authors have described that an oversized device is a risk factor for the occurrence of complete AVB in patients with the perimembranous VSD. We believe that case selection is vitally important in this complication and we did not use oversized devices in our patients. Some authors have suggested that the use of steroids could be helpful to avoid complete AVB and pacemaker implantation.^20^^-^^22^ We did not use steroids for the prevention of pacemaker implantation in our patients, and need for pacemaker implantation in our study population occurred 2 weeks later. Nonetheless, we should monitor complete AVB as a late event occurring in postprocedural phases during the follow-up period. Complete AVB may occur in completely asymptomatic patients. The occurrence of complete AVB has been reported in patients less than 5 years of age at the time of the procedure. Therefore, the percutaneous closure of the perimembranous VSD in young subjects must be carefully suggested to the patients and their family due to the challenging nature of this technique and the risk of complete AVB. At follow-up, patients should be routinely monitored via ECG in every visit and with the ECG Holter if necessary.

Overall, in our study, no early and mid-term major complications such as mortality, device embolization, arterial complications, hemolysis, and endocarditis occurred. We had one patient with complete AVB, which ranks among the lowest range in the literature. The results of this study demonstrated the efficacy of the transcatheter closure of the perimembranous VSD with the Amplatzer VSD Occluder both during early and intermediate follow-up periods.

The most notable limitation of the present study is its mid-term follow-up duration; longer follow-up periods are required in future studies.

## Conclusion

The mid-term follow-up of our pediatric patients showed that the VSD closure with the Amplatzer VSD Occluder was a very safe and effective method for the management of medium to large VSDs. The meticulous selection of patients and devices is essential for reducing complications, especially complete AVB. Complete ABV must be monitored as a late event during long-term follow-up periods. In our study population, the left ventricular dimensions returned to normal. In addition, growth returned to the normal pattern in the patients with failure to thrive. However, there is always the possibility of the occurrence of some complications. More in-depth evaluation of this method with a view to reducing the risk of complete AVB requires further studies with long-term follow-up. 

## Notes:

***This paper should be cited as:*** Ghaderian M, Merajie M, Mortezaeian H, Aarabi Moghadam MY, Shah Mohammadi A. Mid-term Follow-up of the Transcatheter Closure of Perimembranous Ventricular Septal Defects in Children Using the Amplatzer. J Teh Univ Heart Ctr 2015;10(4):182-187.

## References

[1] Hoffman JI, Rudolph AM (1965). The natural history of ventricular septal defects in infancy. Am J Cardiol.

[2] Hoffman JI (1995). Incidence of congenital heart disease: I. Postnatal incidence. Pediatr Cardiol.

[3] Tikanoja T (1995). Effect of technical development on the apparent incidence of congenital heart disease. Pediatr Cardiol.

[4] Du ZD, Roguin N, Barak M, Bihari SG, Ben-Elisha M (1996). High prevalence of muscular ventricular septal defect in preterm neonates. Am J Cardiol.

[5] Waight DJ, Cao QL, Hijazi ZM (2000). Closure of patent foramen ovale in patients with orthodeoxia-platypnea using the amplatzer devices. Catheter Cardiovasc Interv.

[6] Thanopoulos BD, Tsaousis GS, Konstadopoulou GN, Zarayelyan AG (1999). Transcatheter closure of muscular ventricular septal defects with the amplatzer ventricular septal defect occluder: initial clinical applications in children. J Am Coll Cardiol.

[7] Lin A, Mahle WT, Frias PA, Fischbach PS, Kogon BE, Kanter KR, Kirshbom PM (2010). Early and delayed atrioventricular conduction block after routine surgery for congenital heart disease. J Thorac Cardiovasc Surg.

[8] Butera G, Carminati M, Chessa M, Piazza L, Micheletti A, Negura DG, Abella R, Giamberti A, Frigiola A (2007). Transcatheter closure of perimembranous ventricular septal defects: early and long-term results. J Am Coll Cardiol.

[9] Predescu D, Chaturvedi RR, Friedberg MK, Benson LN, Ozawa A, Lee KJ (2008). Complete heart block associated with device closure of perimembranous ventricular septal defects. J Thorac Cardiovasc Surg.

[10] Gu X, Han YM, Titus JL, Amin Z, Berry JM, Kong H, Rickers C, Urness M, Bass JL (2000). Transcatheter closure of membranous ventricular septal defects with a new nitinol prosthesis in a natural swine model. Catheter Cardiovasc Interv.

[11] Hijazi ZM, Hakim F, Haweleh AA, Madani A, Tarawna W, Hiari A, Cao QL (2002). Catheter closure of perimembranous ventricular septal defects using the new Amplatzer membranous VSD occluder: initial clinical experience. Catheter Cardiovasc Interv.

[12] Chessa M, Carminati M, Butera G, Bini RM, Drago M, Rosti L, Giamberti A, Pomè G, Bossone E, Frigiola A (2002). Early and late complications associated with transcatheter occlusion of secundum atrial septal defect. J Am Coll Cardiol.

[13] Kidd L, Driscoll DJ, Gersony WM, Hayes CJ, Keane JF, O'Fallon WM, Pieroni DR, Wolfe RR, Weidman WH (1993). Second natural history study of congenital heart defects. Results of treatment of patients with ventricular septal defects. Circulation.

[14] Fu YC, Bass J, Amin Z, Radtke W, Cheatham JP, Hellenbrand WE, Balzer D, Cao QL, Hijazi ZM (2006). Transcatheter closure of perimembranous ventricular septal defects using the new Amplatzer membranous VSD occluder: results of the U.S. phase I trial. J Am Coll Cardiol.

[15] Pinto RJ, Dalvi BV, Sharma S (2006). Transcatheter closure of perimembranous ventricular septal defects using amplatzer asymmetric ventricular septal defect occluder: preliminary experience with 18-month follow up. Catheter Cardiovasc Interv.

[16] Masura J, Gao W, Gavora P, Sun K, Zhou AQ, Jiang S, Ting-Liang L, Wang Y (2005). Percutaneous closure of perimembranous ventricular septal defects with the eccentric Amplatzer device: multicenter follow-up study. Pediatr Cardiol.

[17] Pedra CA, Pedra SR, Esteves CA, Pontes SC Jr, Braga SL, Arrieta SR, Santana MV, Fontes VF, Masura J (2004). Percutaneous closure of perimembranous ventricular septal defects with the Amplatzer device: technical and morphological considerations. Catheter Cardiovasc Interv.

[18] Holzer R, de Giovanni J, Walsh KP, Tometzki A, Goh T, Hakim F, Zabal C, de Lezo JS, Cao QL, Hijazi ZM (2006). Transcatheter closure of perimembranous ventricular septal defects using the amplatzer membranous VSD occluder: immediate and midterm results of an international registry. Catheter Cardiovasc Interv.

[19] Yip WC, Zimmerman F, Hijazi ZM (2005). Heart block and empirical therapy after transcatheter closure of perimembranous ventricular septal defect. Catheter Cardiovasc Interv.

[20] Walsh MA, Bialkowski J, Szkutnik M, Pawelec-Wojtalik M, Bobkowski W, Walsh KP (2006). Atrioventricular block after transcatheter closure of perimembranous ventricular septal defects. Heart.

[21] Butera G, Gaio G, Carminati M (2009). Is steroid therapy enough to reverse complete atrioventricular block after percutaneous perimembranous ventricular septal defect closure?. J Cardiovasc Med (Hagerstown).

[22] Erdem S, Kizlltaş A, Küçükosmanoğlu O, Ozbarlas N (2012). Temporary atrioventricular complete block that develops following the transcatheter closure of ventricular septal defect. Turk J Pediatr.

